# The differences in health-related quality of life between younger and older adults and its associated factors in patients with type 2 diabetes mellitus in Indonesia

**DOI:** 10.1186/s12955-021-01756-2

**Published:** 2021-04-16

**Authors:** Yunita Sari, Atyanti Isworo, Arif Setyo Upoyo, Agis Taufik, Rahmi Setiyani, Keksi Girindra Swasti, Haryanto Haryanto, Saldy Yusuf, Nasruddin Nasruddin, Ridlwan Kamaluddin

**Affiliations:** 1 Department of Nursing, Universitas Jenderal Soedirman, Purwokerto, Indonesia; 2 Department of Medical Surgical Nursing, STIK Muhammadiyah Pontianak, Pontianak, Indonesia; 3 Faculty of Nursing, Hasanuddin University, Makassar, Indonesia; 4 Department of Medical Laboratory Science, Faculty of Nursing and Health Sciences, Universitas Muhammadiyah Semarang, Semarang, Indonesia

**Keywords:** Diabetes mellitus, Quality of life, Older younger adults, Predictor

## Abstract

**Background:**

It is well known that diabetes mellitus (DM) affects health-related quality of life (HRQOL) in both younger (aged 18–64 years) and older adults (aged ≥ 65 years). However, to date, no study has compared HRQOL and its predictors between younger and older adults with DM in Indonesia. Such a comparison is important because the results can guide nurses and clinicians to establish evidence-based educational programs that are specific and suitable for patients. Therefore, the aim of this study was to investigate the difference in HRQOL and its predictors in younger and older adults with DM in Indonesia.

**Methods:**

A cross-sectional study was conducted on 641 patients with type 2 diabetes mellitus (T2DM) who were recruited via simple random sampling from 16 primary health centers in Banyumas Regency, Indonesia. A self-administered questionnaire containing the Summary of Diabetes Self-Care Activities, the DDS17 Bahasa Indonesia, the Beck Depression Inventory II, the Self-Efficacy for Diabetes Scale, the Family APGAR, and the 36-item Short-Form Health Survey was used to measure diabetes self-management (DSM), diabetes distress (DD), depression, self-efficacy, family support, and HRQOL, respectively. Independent t-tests were used to compare the physical component summary (PCS) and mental component summary (MCS) scores between younger and older adults with T2DM. Hierarchical multiple regression analyses were used to examine the factors associated with HRQOL in both groups.

**Results:**

PCS scores were significantly different between the two groups. Older adults reported lower PCS scores than younger adults. No differences between the two groups were observed in the MCS scores. The hierarchical multiple regression analysis showed that level of education, employment status, number of diabetes-related complications, DSM, DD, depression, and self-efficacy were significant predictors of HRQOL in younger adults, while income, depression, DD, and self-efficacy were significant predictors of HRQOL in older adults. DD was the strongest predictor of HRQOL in younger adults, and depression was the strongest predictor in older adults.

**Conclusion:**

Older adult patients had lower PCS scores than younger adult patients. This study is the first to show that the predictors of HRQOL differ between younger and older adults with T2DM. It provides insights for nurses and clinicians in Indonesia to establish evidence-based, age-specific educational programs.

## Background

The number of patients with diabetes mellitus (DM) is increasing at an alarming rate. Global cases increased by 211 million between 2000 and 2013 [1, 2]. It is predicted that there will be 210 million new cases of DM between 2013 and 2035 [1]. It is commonly assumed that type 2 diabetes mellitus (T2DM) mainly affects older adults. However, data demonstrate that among the 382 million individuals with DM in 2013, the greatest number of patients were younger adults, and, of 5.1 million deaths due to DM, half were younger adults [3, 4].

The number of patients with DM is also increasing in Indonesia. Indonesia has the seventh largest number of patients with DM in the world [3], with nearly 11 million adults having been diagnosed with T2DM. The prevalence of DM in younger and older adults in Indonesia is similar, namely, 4.48% and 5.33%, respectively [5]. Recently in Indonesia, the incidence of T2DM in younger adults has begun to rise [6, 7].

DM has a significantly negative impact on health-related quality of life (HRQOL) in patients, either directly or because of its complications [8, 9]. Additionally, patients with DM tend to have poor HRQOL, especially in regard to physical and psychological functions [10]. Therefore, an assessment of the HRQOL of patients with DM is important because it can help to monitor treatment guidelines to avoid serious consequences. It will also identify individuals with poor HRQOL and predictors that could guide nurses and clinicians to establish evidence-based educational programs that are specific to and suitable for patients. A program based on predictors of HRQOL is important, especially in a developing country such as Indonesia, where resources are limited.

Among demographic variables, age is the most commonly reported predictor of HRQOL. In general, older adults have a worse HRQOL compared with younger adults [11]; however, this remains unclear in patients with DM. A study in patients with DM conducted between 2000 and 2020 found no difference in HRQOL between older and younger adults based on physical component summary (PCS) and mental component summary (MCS) scores [12]. However, this study was conducted in developed countries and, therefore, is not relevant to a developing country such as Indonesia. Additionally, no study has compared HRQOL between younger (aged 18–64 years) and older adults (aged ≥ 65 years) with DM in a developing country, for example, Indonesia. Therefore, difference in HRQOL between younger and older adults with DM is still unclear. Compared to those who live in developed countries, residents of developing countries usually have a lower economic status, a less healthy lifestyle, fewer resources, and lower quality of health care services [13]. These factors, taken alongside the consequences of aging and DM disease progression, allowed us to hypothesize that HRQOL in patients with DM in older adults would be lower than that of younger adults in Indonesia. Because no study has compared HRQOL between younger and older adults with DM in Indonesia, the main aim of this study was to investigate the differences in HRQOL between younger and older adults with DM in this country.

The variables that have been demonstrated to affect HRQOL in patients with DM are diabetes self-management (DSM), diabetes distress (DD), depression, self-efficacy, and family support [10, 14–17]. Although the predictors of HRQOL in patients with DM have been studied, it remains unclear whether they apply to both younger and older adult patients. If data on the differences in the predictors in both groups were available, it would be possible to establish evidence-based educational programs for improving HRQOL in patients with DM that were specific and suitable for each population. To effectively improve HRQOL, the predictors of HRQOL should be examined separately in younger and older adults. Therefore, the second aim of this study was to investigate the difference in the predictors of HRQOL between younger and older adults with DM.

## Methods

### Research participants and data collection

A cross-sectional study was conducted on patients with T2DM who were recruited via simple random sampling from 16 primary health centers in Banyumas Regency, Indonesia. The sample size was calculated using a 95% confidence level, prevalence of 13.4%, and absolute precision of 3%. Considering the response rate of 77.4% based on our previous study (unpublished), the total sample size calculated was 641 patients. The inclusion criteria were patients aged 18 or over who had been diagnosed with T2DM by their physician and were willing to sign the informed consent form. The exclusion criteria were patients with physical disabilities, cognitive or neurological impairments, or critical or advanced complications. The clinical data of patients were extracted from their medical records. The selection of a random samples was conducted by using a computer generated random sample (RAND function in Excel) to select 641 patients out of 1747 (total patients of 16 primary health centers). The ethical research committee at the Faculty of Health Sciences, Purwokerto, Indonesia, approved this study (059/KEPK/II/2020 (11 February 2020).

### Demographic and clinical information

Demographic and clinical variables related to HRQOL were assessed and studied. Variables were age, gender, marital status, level of education, employment status, income, body mass index (BMI), duration of DM, smoking status, number of diabetes-related complications, fasting blood glucose, presence of hypertension, and type of DM medication.

### Research instruments

#### Health-related quality of life

HRQOL was measured using the 36-item Short-Form Health Survey (SF-36), one of the most widely used questionnaires for assessing HRQOL. Other studies have demonstrated that the SF-36 has high validity and reliability [18–20] and it has been validated in Indonesia. Cronbach’s alpha of the Indonesian version of the SF-36 is satisfactory (higher than 0.7) [21]. The questionnaire is composed of eight domains (physical functioning, role limitations related to physical health problems, bodily pain, general health, vitality, social functioning, role limitations related to mental health, and mental health). These domains were scored from 0 to 100 and were clustered into PCS and MCS measures [22, 23], which were transformed into T-scores and normalized to the general population in the United States (mean = 50, standard deviation = 10) [20].

#### Diabetes self-management

DSM was assessed using a summary of the diabetes self-care activities measure (SDSCA). This multidimensional instrument that assesses patient behavior in DSM was developed by Toobert et al. [24] and has been widely used in other countries [25–27]. The SDSCA is composed of a core set of 11 items and 14 extended items. The core items assess diet, exercise, blood-glucose testing, foot care, and smoking [24], while the extended items assess self-care recommendation, diet, medication, foot care, and smoking [24]. Possible responses range from 0 to 7, according to the number of days patients performed self-care over the past week. A higher score means better self-care management. One study demonstrated the content validity of the SDSCA as being 0.83 and Cronbach’s alpha as being 0.69 [27]. The Indonesian version of the SDSCA showed a Cronbach’s alpha of 0.72 [28].

### Diabetes distress

Diabetes-related emotional distress was assessed using DDS17 Bahasa Indonesia, which is an Indonesia version of the Diabetes Distress Scale questionnaire [29]. This scale contains four domains including interpersonal distress, emotional burden, physician-related distress, and regimen-related distress. There are 17 items on the scale, with each item being scored from 1 (not a problem) to 6 (a very serious problem). The total scores of the 17 items ranged from 17 (not a problem) to 102 (a very serious problem). This scale had a Cronbach’s alpha between 0.78 and 0.83 [29].

### Depression

The Beck Depression Inventory II (BDI II) is an instrument for assessing the severity of subjective depressive symptoms [30]. Composed of emotional, cognitive, motivational, and physiological items, the BDI II is one of the most widely used measures of depression. This scale has been validated in many countries, and had a Cronbach’s alpha between 0.86 and 0.93 [31–33]. The Indonesian version of the BD II had a Cronbach’s alpha between 0.74 and 0.81 [34]. The questionnaire is composed of 21 statements, with each statement being scored from 0 to 3. The total score, up to a maximum of 63, is obtained by adding each score for the 21 items. Higher scores indicate greater depression.

### Self-efficacy

Self-efficacy was assessed using the self-efficacy for diabetes scale (SES) from the Stanford Patient Education Research Centre [35]. This questionnaire assesses how confident patients are in performing activities related to their diabetes, including diet management, exercise, blood glucose control, and illness management. The scale is composed of eight Likert-type scale items that range from 1 (not at all confident) to 10 (totally confident). This scale had a Cronbach’s alpha of 0.82 [35] and the Indonesian version of SES showed similar Cronbach’s alpha value (0.82) [36]. A score equal to or greater than the mean can be categorized as good self-efficacy [35].

### Family support

Family support was assessed using the Family APGAR, which has been widely used to measure perceived family support in five domains: adaptation (A), partnership (P), growth (G), affection (A), and resolve (R) [37]. Other studies have demonstrated the tool’s validity and reliability to be satisfactory [37, 38]. A previous study showed that the Indonesian version of this questionnaire had a Cronbach’s alpha of 0.83 [39]. Questions are ranked from 0 (hardly ever) to 2 (almost always). The highest possible overall score is 10, with a score of between 8 and 10 indicating a highly functional family, and a score below 8 indicating a dysfunctional family [40].

### Statistical analysis

A statistical evaluation was conducted using SPSS version 22.0 (SPSS Inc., Chicago, IL, USA).

Descriptive statistics (mean, standard deviation, and percentage) were used to describe the demographic and clinical characteristics of the patients. Mean, and standard deviation were calculated for continuous data, and percentage values were calculated for discrete data. To identify whether the data were normally distributed, visual inspections of the histograms were performed and the Kolmogorov–Smirnov test was used. We found that the data were normally distributed. To identify differences between younger and older adults in the demographics and clinical characteristics, independent t-tests were used to compare interval variables and chi-square tests were used to compare categorical variables. Differences in HRQOL domains were analyzed using an independent t-test. Separate models were constructed to identify the predictors of HRQOL in younger and older adults. To identify the predictors of HRQOL in younger and older adults, a hierarchical multiple regression analysis was used. In the first block, DSM, DD, depression, self-efficacy, family support, and HRQOL were entered in the analysis. In the second block, demographic variables were jointly entered into the analysis. In the third block, the clinical data were jointly entered into the analysis. Tolerance and variance inflation factors were examined to detect multicollinearity. Tolerance values of less than 0.20 and variance inflation factor values higher than 5 indicated a multicollinearity problem [41–43].

## Results

### Demographic characteristics

The demographics and clinical characteristics of the patients are presented in Table 1. A total of 641 patients were included with a response rate of 100%. Younger adult patients had a lower level of education (*p* = 0.032), lower income (*p* = 0.032), higher BMI (*p* < 0.001), shorter duration of DM (*p* < 0.01), fewer diabetes-related complications (*p* = 0.009), and higher fasting blood glucose levels than older patients (*p* < 0.01). There were no differences between the groups in employment status, smoking status, presence of hypertension, or type of DM medication used. These demographics and clinical characteristics were controlled in the subsequent analysis.

**Table 1:** Subject characteristics according to age

Characteristic	Younger adults	Older adults	*p* value
	(18–64) years	(≥ 65) years	
	(N = 435)	(N = 206)	
	Mean (± SD) or n (%)	Mean (± SD) or n (%)	
*Age*	55. 32 ± 6.80	70.00 ± 4.60	
*Gender*			0.008*
Male	86 (19.77)	60 (29.13)	
Female	349 (80.23)	146 (70.87)	
*Marital status*			< 0.001*
Single/never married	8 (1.84)	2 (0.97)	
Married	375 (86.21)	139 (67.48)	
Widowed	52 (11.95)	65 (31.55)	
*Level of education*			0.032*
Illiterate	32 (7.36)	11 (5.34)	
Elementary school	271 (62.30)	105 (50.98)	
Junior High School	61 (14.02)	39 (18.94)	
Senior High School	49 (11.26)	27 (13.10)	
College or higher	22 (5.06)	24 (11.65)	
*Employment status*			0.219
Employed	186 (42.76)	92 (44.66)	
Unemployed	249 (57.24)	114 (55.34)	
*Income*			0.032*
Low income (less than USD 138 per month)	389 (89.43)	171(83.00)	
Middle income (USD 138–177 per month)	36 (8.28)	31(15.06)	
High income (higher than USD 177 per month)	10 (2.29)	4 (1.94)	
*BMI*	24.32 ± 4.70	22.90 ± 4.20	< 0.001*
*Duration of DM*			< 0.001*
Less than 1 year	49 (11.26)	10 (4.85)	
1–5 years	238 (54.71)	99 (48.05)	
6–10 years	96 (22.07)	50 (24.27)	
More than 10 years	52 (11.96)	47 (22.81)	
*Smoking status*			0.295
Yes	25 (5.75)	16 (7.77)	
No	410 (94.25)	190 (92.23)	
*Hypertension*			0.70
Yes	278 (63.91)	143 (69.42)	
No	157 (36.09)	63 (30.58)	
*Number of diabetes related complications*			0.009*
No complications	191(43.90)	66 (32.03)	
One complication	157 (36.10)	93 (45.14)	
Two or more complications	87 (20.0)	47 (22.81)	
*Fasting blood glucose (mg/dl)*	116 ± 8.90	104 ± 42.70	0.001*
*Type of DM medication*			0.526
No medication	26 (5.98)	17 (8.25)	
Insulin	375 (86.20)	177 (85.93)	
Oral medication	15 (3.45)	4 (1.94)	
Oral medication and insulin	19 (4.37)	8 (3.88)	

Data are expressed as group mean (SD) or percentage

Classification of income was according to minimum regional wage

*DM* diabetes mellitus

*p < 0.05

### HRQOL

Controlling for demographic and clinical characteristics, the PCS score was significantly lower in older adults than in the younger adults (Table 2). The analysis of SF-36 sub-dimensions showed significant differences in physical function and role limitation due to physical problems: older adults, compared with younger adults, reported lower physical function (*p* < 0.001) and greater role limitation due to physical problems than younger adults (*p* < 0.001). After controlling for demographics and clinical characteristics, there was no significant difference in MCS scores between younger and older adults. The SF-36 sub-dimension analysis found that older adults had lower social function (*p* < 0.001) than younger adults.

**Table 2:** Comparison of the PCS and MCS between younger and older adults

Variables	Younger adults (18–64) years	Older adults (≥ 65) years	*t*	*p* value
	(N = 435)	(N = 206)		
	Mean (± SD) or n (%)	Mean (± SD) or n (%)		
*PCS*	49.90 ± 5.52	47.74 ± 5.48	− 4.626	< 0.001*
Physical function	83.90 ± 16.30	73.60 ± 17.10	− 7.348	< 0.001*
Bodily pain	56.30 ± 24.80	58.20 ± 21.60	− 1.114	0.266
General health	65.50 ± 10.00	64.50 ± 10.40	0.984	0.325
Role limitation due to physical problems	74.70 ± 24.10	68.90 ± 23.50	− 2.868	< 0.001*
*MCS*	38.16 ± 3.25	38.06 ± 3.53	− 0.320	0.749
Social function	50.70 ± 12.10	44.40 ± 12.60	− 0.659	< 0.001*
Vitality	61.50 ± 17.90	60.60 ± 16.40	0.092	0.503
Mental health	44.25 ± 15.00	43.90 ± 14.10	− 0.318	0.750
Role limitation due to emotional problems	77.20 ± 24.30	75.10 ± 25.50	− 1.013	0.318

Data are expressed as mean ± standard deviation

*PCS* physical component summary, *MCS* mental component summary

*p < 0.05

### Predictors of HRQOL in younger adults

The predictors of HRQOL in younger adults are shown in Table 3. In the first block, DSM, DD, depression, family support, and self-efficacy were entered into the analysis. The analysis showed that DSM, DD, depression, and self-efficacy were associated with HRQOL. Model 1 accounted for 26.4% of the variance observed in HRQOL. In the second block, the demographic variables were jointly entered into the analysis. DSM, DD, depression, and self-efficacy remained significant predictors for HRQOL. In the second block, the only demographic factor that became a significant predictor was the level of education. In the third block, clinical data were jointly entered into the analysis. DSM, DD, depression, self-efficacy, level of education, employment status, and number of diabetes-related complications were significant predictors of HRQOL (F = 11.63, *p* < 0.001). These variables accounted for 29.3% of the variance observed in HRQOL. The tolerance values of the third model ranged from 0.783 to 0.924 (> 0.20), and the variance inflation factor ranged from 1.023 to 1.373 (≤ 5), indicating that the model did not exhibit multicollinearity problems. Therefore, a regression model was deemed appropriate.

**Table 3:** Predictors of HRQOL in younger adults patients with DM

Variables	β	*t*	*p value*
*Steps 1*			
Constant		14.895	< 0.001
DSM	0.155	3.537	< 0.001*
DD	− 0.295	− 6.236	< 0.001*
Depression	− 0.153	− 3.270	0.001*
Self-efficacy	0.175	3.974	< 0.001*
Family support	0.035	0.831	0.407
Adjusted R^2^ = 0.264; F = 32.38 and *p* < 0.001			
*Step 2*			
Constant		14.537	< 0.001
DSM	0.146	3.327	0.001*
DD	− 0.303	− 6.371	< 0.001*
Depression	− 0.144	− 3.102	0.002*
Self-efficacy	0.162	3.675	< 0.001*
Family support	0.036	0.867	0.386
Gender	− 0.009	− 0.200	0.842
Marital status	− 0.040	− 0.957	0.339
Level of education	0.119	2.737	0.006*
Employment status	0.088	1.856	0.064
Income	0.076	1.802	0.072
Adjusted R^2^ = 0.281; F = 18.04 and *p* < 0.001			
*Step 3*			
Constant		9.981	< 0.001
DSM	0.122	2.765	0.006*
DD	− 0.288	− 5.827	< 0.001*
Depression	− 0.118	− 2.498	0.013*
Self-efficacy	0.133	2.974	0.003*
Family support	0.035	0.830	0.407
Gender	− 0.011	− 0.234	0.815
Marital status	− 0.033	− 0.805	0.421
Level of education	0.097	2.228	0.026*
Employment status	0.094	1.991	0.047*
Income	0.081	1.910	0.057
BMI (kg/m^2^)	0.044	1.051	0.294
Duration of DM	− 0.070	− 1.630	0.104
Smoking status	0.067	1.555	0.121
Number of diabetes-related complications	− 0.106	− 2.364	0.019*
Fasting blood glucose	− 0.051	− 1.252	0.211
Hypertension	0.007	0.147	0.883
Type of DM medication	0.047	1.127	0.260
Adjusted R^2^ = 0.293; F = 11.64, and *p* < 0.001			

In the first block, DSM, DD, depression, self-efficacy, and family support were entered into the analysis. In the second block, demographical factors were jointly entered into the analysis. In the third block, clinical data were jointly entered

*DSM* diabetes self-management, *DD* diabetes distress, *BMI* body mass index

*p < 0.05

### Predictors of HRQOL in older adult patients

Predictors of HRQOL in older adults are shown in Table 4. In the first block, DD, depression, and self-efficacy were significant predictors of HRQOL. Model 1 accounted for 31.4% of the variance observed in HRQOL. In the second block, DD, depression, and self-efficacy remained significant predictors of HRQOL. Income was the only significant demographical factor in the second block. Model 2 accounted for 33.6% of the variance observed in HRQOL. In the third block, DD, depression, self-efficacy, and income were significant predictors of HRQOL. These variables accounted for 32.1% of the variance observed in HRQOL. The tolerance values of the third model ranged from 0.673 to 0.924 (> 0.20), and the variance inflation factor ranged from 1.083 to 1.486 (≤ 5), indicating that the model did not exhibit multicollinearity. Therefore, a regression model was deemed appropriate.

**Table 4:** Predictors of HRQOL in older adults patients with DM

Variables	β	*t*	*p value*
*Steps 1*			
Constant		12.720	< 0.001
DSM	0.030	0.487	0.627
DD	− 0.204	− 3.197	0.002*
Depression	− 0.346	− 5.314	< 0.00*
Self-efficacy	0.219	3.299	0.001*
Family support	0.007	0.110	0.912
Adjusted R^2^ = 0.314; F = 19.62 and *p* < 0.001			
*Step 2*			
Constant		11.440	< 0.001
DSM	0.064	1.039	0.300
DD	− 0.210	− 3.324	0.001*
Depression	− 0.356	− 5.468	< 0.001*
Self-efficacy	0.211	3.215	0.002*
Family support	0.005	0.081	0.935
Gender	− 0.039	− 0.604	0.546
Marital status	− 0.057	− 0.933	0.352
Level of education	0.011	0.176	0.861
Employment status	0.083	1.246	0.214
Income	0.144	2.280	0.024
Adjusted R^2^ = 0.336; F = 11.27 and *p* < 0.001			
*Step 3*			
Constant		7.707	< 0.001
DSM	0.062	0.989	0.324
DD	− 0.207	− 3.114	0.002*
Depression	− 0.365	− 5.431	< 0.001*
Self-efficacy	0.202	2.998	0.003*
Family support	− 0.006	− 0.098	0.922
Gender	− 0.021	− 0.292	0.770
Marital status	− 0.055	− 0.856	0.393
Level of education	0.001	0.019	0.985
Employment status	0.090	1.308	0.192
Income	0.140	2.138	0.034*
BMI (kg/m^2^)	− 0.050	− 0.816	0.416
Duration of DM	− 0.005	− 0.090	0.928
Smoking status	− 0.042	− 0.660	0.510
Number of diabetes-related complications	0.013	0.214	0.831
Fasting blood glucose	− 0.043	− 0.715	0.475
Hypertension	− 0.013	− 0.194	0.846
Type of DM medication	0.053	0.840	0.402
Adjusted R^2^ = 0.321; F = 6.64 and *p* < 0.001			

In the first block, DSM, DD, depression, self-efficacy, and family support were entered into analysis. In the second block, demographical factors were jointly entered into analysis. In the third block, clinical data were jointly entered

*DSM* is diabetes self-management, *DD* is diabetes distress, *BMI* is body mass index

## Discussion

This relatively large-scale cross-sectional study of patients with T2DM was the first study conducted in Indonesia to compare HRQOL between younger and older adults and its associated factors in patients with T2DM. The core finding of this study was that the predictors of HRQOL in younger adults were neither similar to nor different from those in older adults. We found seven predictors of HRQOL in younger adults, and four predictors of HRQOL in older adults. The predictors of HRQOL in younger adults were level of education, employment status, number of diabetes-related complications, DSM, DD, depression, and self-efficacy. The predictors of HRQOL in older adults were income, depression, DD, and self-efficacy. We also found DD to be a stronger predictor than depression in younger adults and depression to be a stronger predictor than DD in older adults. This is the first study to show that predictors of HRQOL in younger and older adults are not similar. This study provides new knowledge for the literature and evidence for nurses and clinicians to establish specific interventions to improve age-specific HRQOL for patients with T2DM in Indonesia.

In this study, older patients had a lower PCS scores than younger patients. Our findings differed from those of Trief et al. [12], who found the PCS level to be the same between both groups. The difference in PCS scores might be because in Indonesia, older adults tend to engage in lower levels of physical activity and have more diabetes complications than younger adults. Thus, we can see the importance of designing a program to improve PCS in older adults in Indonesia.

Somewhat surprisingly, our MCS results differed from our hypothesis. We found no difference between the MCS of younger and older adults. We also found that MCS scores in both younger and older adults were below those of the general population. This could be explained by the fact that all participants in our study tended to suffer from DD and depression. Most of the younger adults in this study had had DM for between one and five years. Thus, they might still be adapting to diabetes management. Another study showed that difficulty in following diabetes management could result in DD in adult patients [44]. In older adults, depression might result from the aging process and complications related to DM. Another possible reason is that most of the older adults in this study had a deceased spouse. Living alone is a risk factor for lower mental component-related HRQOL in older adults since no emotional support is given by a spouse [45–48]. According to Weiss’s attachment theory, having a spouse can prevent individuals from suffering emotional loneliness that affects mental health [49]. Further research to explore the specific cause of DD and depression in both groups is necessary.

Our study found that both DD and depression were predictors of HRQOL, however, in younger adults, DD was a stronger predictor than depression, and in older adults, depression was a stronger predictor than DD. Many other studies have found that depression and DD affect HRQOL [50–52]. However, according to our review of the literature, this study was the first to show that when comparing DD and depression, DD was a stronger predictor for HRQOL in younger adults, and depression was a stronger predictor in older adults. DD refers to emotional distress related to living with and managing diabetes [53]. Many of the younger adults in this study had DM for less than five years, and many of the older adults had DM for more than five years. Therefore, we can assume that older adults knew how to manage the disease more effectively, as indicated by their ability to control their blood glucose better than the younger adults. The inability to manage DD might cause DD to become a stronger predictor in younger adults. Many younger adult patients in our study reported suffering mild depression, while many older adults reported moderate or severe depression. One study found that a depressive state has to be sufficient in intensity and duration to affect HRQOL [54]. This could be why, in younger adults, depression was not a stronger predictor than DD. The possible reasons for such differences require further research.

In this study, DSM was one of the predictors of HRQOL in younger adults but not in older adults. Our findings were not in accordance with those of Huang and Hung [55] who reported that DSM was also a predictor of HRQOL in older adults. A possible explanation for the difference is that older adults in Indonesia might perceive DSM to be a routine activity. Because of this they may perform diabetes self-care management only to maintain physical and emotional homeostasis, and therefore, it may not have significant effects on HRQOL.

Self-efficacy was found to be a predictor in both younger and older adults. DM patients with good self-efficacy means that they have confidence in their abilities to manage diabetes and influence disease outcomes [56]. Our study showed that individuals with higher levels of self-efficacy had better HRQOL. These results corresponded with those of Bowen et al. [16] who showed that self-efficacy was a predictor of HRQOL in patients with DM. Thus, a program to improve self-efficacy is necessary for both younger and older adults in Indonesia.

In this study, demographic variables that were found to be predictors of better HRQOL in younger adults were level of education, employment status, and number of diabetes-related complications. These results correspond with those of previous studies [57–60]. We also found that the main sociodemographic predictor of HRQOL in older adults was income. This finding supported those of two other studies that found older adults with a higher income to have a better HRQOL than those with a lower income [61, 62].

With this study, we identified predictors of HRQOL in younger and older T2DM patients in Indonesia. This study adds to the growing body of evidence that the predictors of HRQOL in younger and older patients are different. Based on our study findings, several actions should be taken. First, government and health clinicians should pay more attention to the physical and mental health of diabetic patients since this can affect HRQOL. Second, nurses and clinicians should create educational programs designed to improve DSM and patients’ mental health in order to be able to manage DD and depression. Future studies are needed to evaluate the effectiveness of the implementation of educational programs in improving HRQOL.

This study had several limitations. First, since it was a cross-sectional study, no causal relationship could be drawn between the variables. Second, the study was conducted in Banyumas Regency and thus any extrapolation of the results to the rest of the Indonesian population should be carried out with caution. Third, we used the widely used QOL scale (SF-36] rather than a specific scale for HRQOL. Thus, there is a need for future research using a more specific scale. Finally, most patients in our study were female. While this could be perceived as a limitation, it does, however, represent the situation in Indonesia, since most DM patients in Indonesia are female.

Despite its limitations, our study also has strengths. First, it involved a large number of patients with DM in Indonesia. We had a high response rate and were able to form a representative sample regarding sociodemographics. Second, we used scales that have been validated in Indonesian settings. Third, this study was the first to examine the differences in HRQOL and its predictors in younger and older adults in T2DM patients in Indonesia. Therefore, the results of this study are critical for nurses and clinicians in Indonesia wanting to establish specific interventions to improve HRQOL in both groups. This study provides a foundation for further empirical studies on intervention methods to improve HRQOL based on the predictors we identified in both younger and older adults. There is a need for a larger, longitudinal study to assess the effects on HRQOL in patients who undergo specially designed programs.

## Conclusion

This study is the first to compare HRQOL between younger and older adults and its associated factors in patients with T2DM in Indonesia. It provides evidence for nurses and clinicians in Indonesia to develop new approaches to improve HRQOL in both younger and older DM patients. The main findings of our study were the predictors of HRQOL in younger adults were neither similar to nor different from those in older adults. We found seven predictors of HRQOL in younger adults, and four predictors of HRQOL in older adults. The predictors of HRQOL in younger adults were level of education, employment status, number of diabetes-related complications, DSM, DD, depression, and self-efficacy, while the predictors of HRQOL in older adults were income, depression, DD, and self-efficacy. The strongest predictor of HRQOL in younger adults was DD, while the strongest predictor of HRQOL in older adults was depression. Nurses and clinicians should design educational programs for patients with DM to improve DSM, as well as to improve mental health to overcome DD and depression.

## Data Availability

The datasets of the current study are available from the corresponding author on request.
